# The Current Landscape of Sacred Moments Research: Reconsidering Meaning, Relationality, and Well‐Being in Health Care

**DOI:** 10.1002/jgf2.70136

**Published:** 2026-05-24

**Authors:** Takashi Watari, Sanjay Saint

**Affiliations:** ^1^ Integrated Clinical Education Center Kyoto University Hospital Kyoto Japan; ^2^ General Medicine Center Shimane University Hospital Izumo Japan; ^3^ Department of Internal Medicine University of Michigan Medical School Ann Arbor Michigan USA; ^4^ Medicine Service, VA Ann Arbor Healthcare System Ann Arbor Michigan USA

A “sacred moment” is a brief period characterized by deep connection between a patient and clinician, or between people engaged in care, accompanied by spiritual emotions such as awe, gratitude, serenity, and joy. The phenomenon of sacred moments was first conceptualized in psychotherapy by Pargament et al. [1] as a relational experience of special meaning that transcends ordinary technical therapeutic exchange. In the United States, the concept has subsequently been extended to acute inpatient care [2], physician well‐being research in internal medicine [3], and radiation oncology [4]. More recently, beyond the Western cultural context, the concept has been explored in Japanese health care [5]. Sacred moments should not be regarded merely as peripheral expressions of religiosity or sentiment. Rather, they are increasingly recognized as a clinically meaningful construct that links patient–clinician relationships, holistic care, professional meaning in work, and prevention of burnout. In this editorial, we discuss five key insights into sacred moments of relevance to general and family medicine.

First, sacred moments are not uncommon in clinical practice. In an exploratory qualitative study of hospitalized patients and health care workers, Quinn et al. [2] found that both patients and clinicians reported experiencing such moments at least once in their lives. In addition, a national survey of U.S. internal medicine physicians by Ameling et al. [3] found that two‐thirds of the respondents had experienced a sacred moment with a patient. An exploratory study from Japan further showed that, after the concept was explained, 85% of clinicians and 75% of patients reported that they had experienced a similar phenomenon [5]. These findings suggest that sacred moments are not a uniquely Western phenomenon but can also arise within the Japanese healthcare environment. Sacred moments may have long been present in everyday clinical work yet remained overlooked because the phenomenon had not been adequately named, conceptualized, or made visible within medicine.

Second, sacred moments may humanize the patient–clinician relationship and deepen the quality of care. In studies of inpatient medicine, these moments are characterized by sincere listening, shared vulnerability, genuine interest in the individual, reassurance, and trust [2, 3, 5]. In qualitative work from radiation oncology, sacred moments have been described as experiences that allow clinicians to see the patient not simply as a case of disease and treatment, but as a whole person, and that they also impart a sense of meaning in the clinician's professional role [4]. This is particularly relevant in the care of patients with terminal illnesses, in whom the clinical value of care lies not only in the accuracy of explanations or the appropriateness of treatment decisions, but also in the capacity to share suffering, uncertainty, and presence. Sacred moments thus provide a framework for redefining quality in health care so that it includes not only technical excellence, but also the quality of human connection.

Third, sacred moments are associated with greater clinician well‐being lower likelihood of burnout. In the national survey of U.S. internal medicine physicians by Ameling et al. [3], physicians who experienced sacred moments several times per year or more had a substantially lower probability of extreme burnout than those who experienced them less frequently. Moreover, discussing such sacred moments with colleagues was associated with a lower risk of burnout [3]. Similarly, research in patients with cancer undergoing radiation suggests that sacred moments may restore the humanity of the patient, enhance clinicians' sense of purpose and job satisfaction, and function as a buffer against burnout [4]. These findings suggest that clinician burnout cannot be adequately understood solely in terms of workplace conditions, excessive workload, or institutional design, but must also be understood in relation to the erosion of meaning in work and the quality of interpersonal relationships. Sacred moments therefore point toward a broader and more complete understanding of professional sustainability in medicine.

Fourth, sacred moments are not merely accidental events but can be intentionally fostered. Quinn et al. [2] identified several elements that support their emergence, including pausing to listen, attending to the individual person, sharing emotion, and creating a safe space for dialogue. Saint et al. [4] identified lack of time to engage with patients as a major barrier to fostering sacred moments, a finding supported by the Japanese study by Sakama et al. [5] that found time to build rapport and trust emerged as a central condition. This study also highlighted the difficulty of translating the concept to Japanese culture using the term “sacred moment,” since the word “sacred” was found to evoke strong religious associations. Collectively, these findings suggest that sacred moments should not be viewed as mysterious events dependent only on individual sensitivity. Rather, they should be understood as clinical phenomena supported by organizational and cultural conditions, including time, continuity, dialogue, kindness, reflective communication, and a culture of psychological safety.

Finally, the concept of sacred moments appears to extend beyond patient–clinician relationships and may also apply to mentee–mentor relationships in medical education. Sacred moments can also occur within mentoring relationships, as illustrated by the example of a junior faculty member who, after the rejection of a research grant, regained self‐efficacy and continued an academic career because of supportive words from a mentor [6]. The core elements of sacred moments—deep listening, shared vulnerability, affirmation, and trust—are equally important in educational relationships. Moments in which learners feel that they are truly understood and genuinely recognized may strongly support psychological safety, a sense of belonging, professional identity formation, resilience, and may prevent disengagement and attrition. In this sense, sacred moments may develop into a useful educational construct linking bedside teaching, mentorship, and faculty development. Currently, the literature on sacred moments in education remains largely conceptual and descriptive. Further qualitative, longitudinal, and interventional research is needed to clarify the conditions under which such moments arise and to determine how they relate to educational outcomes.

In summary, although research on sacred moments is currently limited, the literature to date consistently suggests that this concept is deeply connected to meaning, relationship, purpose, and professional sustainability in health care. Taken together, the five insights discussed herein suggest that sacred moments are neither rare nor incidental. Rather, sacred moments humanize care, support clinician well‐being, can be intentionally fostered, and may extend beyond clinical encounters into medical education and mentoring. Such Sacred moments are thus best understood a meaningful relational construct that can help general and family medicine clinicians remain grounded in positive human interactions. Three possibilities for future research warrant attention. First, the concept of “sacred moments” and the tools used to measure them need to be refined for application in non‐Western settings, including Japan, where linguistic and cultural translation is essential. Second, prospective studies are needed to explore the associations between sacred moments and patient outcomes, clinician burnout, and professional development. Third, interventions that protect temporal space, continuity, reflective conversation, and shared dialogue should be investigated as possible means to increase the occurrence of sacred moments in clinical settings. At a time when efficiency and standardization dominate modern health care, sacred moments remind us that the essence of medicine lies not only in managing disease, but also in accompanying human suffering and sharing meaning. Sacred moments may therefore become recognized as a central concept that bridges whole‐person care, clinician well‐being, and restores humanity to educator‐learner relationships within medical education.

## Author Contributions

**Takashi Watari:** conceptualization, methodology, data curation, project administration, resources, writing – review and editing, writing – original draft. **Sanjay Saint:** conceptualization, data curation, supervision, writing – review and editing, funding acquisition, methodology.

## Funding

The authors have nothing to report.

## Conflicts of Interest

Dr. Takashi Watari is a member of the Editorial Board of JGFM and a co‐author of this article. To minimize bias, he was excluded from all editorial decision‐making related to the acceptance of this article for publication.

## Data Availability

Research data are not shared.
